# Using process drama to explore the causes of dental anxiety in primary-school children

**DOI:** 10.1007/s40368-021-00623-4

**Published:** 2021-04-23

**Authors:** J. F. Tahmassebi, M. Malik, N. Berg, S. Pavitt, K. Gray-Burrows, A. O’Grady

**Affiliations:** 1 Department of Paediatric Dentistry, Faculty of Medicine and Health, School of Dentistry, Leeds School of Dentistry, University of Leeds, Level 6, Worsley Building, Clarendon Way, Leeds, LS2 9LU UK; 2 Leeds School of Dentistry, Leeds, UK; 3 The School of Performance and Cultural Industries, University of Leeds, Leeds, UK; 4 Division of Applied Health and Clinical Translation, Leeds School of Dentistry, Leeds, UK

**Keywords:** Dental anxiety, Drama, Role-play, Fear, Children

## Abstract

**Background:**

Drama and role play can be unlisted as methods to allow children to view problems from a range of different perspectives that may differ from their own experience. Application of drama technique to assess the cause of dental fear and anxiety in a school setting is novel.

**Aim:**

The aim of this study was to engage primary school children in the core investigation via participatory arts methodologies, namely, process drama to gain understanding of the causes of dental anxiety.

**Design:**

Sixty-three children, aged 7–10 years from three primary schools participated in this study. A 90-min drama workshop was carried in each school. The children were encouraged to identify the causes of dental anxiety using key concepts from process drama. The sessions were audio-recorded and transcribed.

**Results:**

Four key concepts emerged: (1) fear of the unknown; (2) unpleasant sensory experience; (3) society’s perception and portrayal of the dentist; and (4) learnt negative associations with the dentist. Within each four key concepts, two sub-themes were identified.

**Conclusions:**

Role-playing and use of drama are a novel application and can reveal a considerable amount of information from the child’s perspective on the cause of dental fear and anxiety.

## Background

Dental fear and anxiety are common problems which can affect people of all ages, but appear to develop mostly in childhood and adolescence (Locker et al. 1999; Tickle et al. 2009). The terms ‘dental fear’ and ‘dental anxiety’ are frequently used interchangeably, and the umbrella term ‘dental fear and anxiety’ (DFA) will be used in this paper.

There is a paucity of systematic reviews estimating the prevalence of dental anxiety in children and adolescents. The systematic review by Klingberg and Broberg measured the prevalence and means score of DFA across a range of measures in children/adolescents (Klingberg and Broberg 2007). In their review, 17 studies were included. They showed the prevalence for DFA range from 5.7 to 19.5% (12 populations) with a mean over all applicable studies of 11.1%. With a wide disparity dependent on the measures employed, Porritt et al. showed that the reliability and validity estimates were good across the most widely used DFA measures, but many had a limited focus on the attributes of anxiety that they assessed (Porritt et al. 2012). Cianetti et al.’s study indicated that at least one child out of ten had a level of DFA that hindered his/her ability to tolerate dental treatment and that the prevalence decreased with increasing age (Cianetti et al. 2017).

Patients with DFA are more likely to avoid dental care (Morgan et al. 2017), which in turn may result in significant deterioration of oral health and can lead to a spiral of increasing dental anxiety (Armfield et al. 2007). The extent to which this is a causal relationship is unknown. An understanding of the full picture underlying the exceptionally complex psychology of dental fear and anxiety is critical in aiding investigators in developing interventions intended to reduce dental anxiety and fear.

The assessment of DFA is complex, and there are broadly three methods used to measure dental anxiety in children. The first method is “behaviour assessment” in which the dental team or researchers are asked to rate both the emotional and behavioural reactions shown by the children during treatment. The second approach is “psychometric assessment” in which the children or one of their parents have to complete a questionnaire, such as the Dental Anxiety Scale (DAS), Modified Child Dental Anxiety Scale (MCDAS) or Children’s Fear Survey Schedule-Dental Subscale (CFSS-DS) questionnaire (Porritt et al. 2012), usually before receiving treatment, to stipulate the level of anxiety associated with different common dental situations. The third technique is “physiological response analysis” in which various factors linked to anxiety are measured, such as heart rate or salivary cortisol level (Porritt et al. 2012). The measuring tools should be quick, relevant for children and their dental experience, and simple to analyse and score (Buchanan 2005). However, there is not yet a standardised method to evaluate dental anxiety and most methods involving questionnaires do not show constant reproducibility and reliability (Welbury et al. 2018).

The DFA assessments are typically measured within a dental surgery, where the child may exhibit more anxiety and fear. The development of dental anxiety in children remains poorly understood. Most studies have relied on parental completion of questionnaires (Tickle et al. 2009). At the inception of this study, there was a dearth of literature on the causes of dental anxiety from a child’s perspective gathered outside the dental setting.

Drama, theatre, and role-playing methods have been used in health promotion programmes in the medical field, but evidence of their effectiveness is limited (Joronen et al. 2008). It is not uncommon for theatre to be employed in the dissemination of health research and for the purposes of public engagement (Rossiter et al. 2008). However, many theatre practitioners working in educational settings actively resist overt message making, preferring instead to encourage audiences to think critically and challenge assumptions about normative behaviours. Process drama is a method of teaching and learning, where both pupils and teacher work in and out of role to explore a problem, situation, theme or issue. Process drama, developed in the 1970s by Dorothy Heathcote, is an established method for engaging children in critical thinking through dramatic play (Heathcote et al. 1991). The work is unscripted but follows a framework set out by the teacher or facilitator. This framework allows pupils to create an imaginary scenario in which they work together to find solutions to ethical, moral or social questions. The use of role play allows children to view problems from a range of different perspectives that may differ from their own. The fictional frame keeps the children one-step removed from the issue at hand, thus providing greater distance from which to view material or content that might be challenging or upsetting.

There is no current literature using drama to involve children outside a dental setting to find out the causes of dental fear and anxiety by involving them in role-play. Therefore, the aim of this study was to engage children in the core investigation via participatory arts methodologies, namely, process drama, to identify the causes of dental anxiety in children.

## Methods

The study was conducted following ethical approval by the Dental Research Ethics Committee of the University of Leeds, reference (090119/JT/268). A convenience sample of five primary schools in the Leeds, UK area were identified through the RAISED in Yorkshire research collaboration. After contacting the head teachers of these primary schools, three schools agreed to take part. Information sheets regarding the study with the consent and assent forms were distributed through the schools to the parents/carers of children aged 7–10 years.

Each school was offered a 90-min workshop for a single class of pupils. The workshop was planned by the research team and delivered in a similar manner and as consistently as possible in each school setting. Each workshop was planned by researcher (AO), Professor in Applied Performance with research expertise in participatory performance and drama, and it was facilitated by AO and another applied theatre specialist (NB) and assisted by one dental student (MM) and one paediatric dental specialist (JFT). At each session, one class teacher was present in each school, accompanied by one teaching assistant. Everyone in the room participated in all activities and there was no external ‘audience’.

The main aim of the workshop was to encourage children to identify the causes of dental anxiety by investigating the topic using key conventions of process drama. The fiction was left open intentionally. Very few clues were given about the central character and no resolution was offered. Instead, the children were presented with a situation, a character and a challenge.

### Establishing the ‘rules of the game’

As the research team were unknown to the pupils, the workshop began with informal introductions around the circle and everyone was provided with a name badge. The facilitator framed the workshop as an opportunity to assist the research team with a ‘problem’. The facilitator told the children they would be working through a story to find out possible solutions to the problem and asked if they would be happy to play different characters along the way. The facilitator explained that she and other members of the research team would also be playing different characters and that they would give clear signals when that was going to happen. Once an agreement had been reached, the workshop began.

### Introducing the topic through small group activity

The first activity functioned as an ice breaker and introduced the topic of dental anxiety in a way that supported the children’s participation from within group discussion. This allowed sharing findings with the whole group, providing the bridge into the next section, where the fictional scenario was explained.

In this introductory activity, children were divided into small groups and were given a set of picture cards in an envelope. Each card depicted a different activity and the children were asked to rank the activities from the most to the least appealing thing to do in the school holidays. The activities included fun things (e.g., go swimming, go to the cinema, ride a bike in the park); mundane, domestic jobs (e.g., hang out the washing, help weed the garden, tidy my bedroom); and some medical-related activities (e.g., have an injection, take off a plaster, visit the dentist). A member of the research team worked with each group to facilitate discussion. The children were then asked to explain their rank order and to articulate to the whole class why they had chosen to put ‘visit the dentist’ in a particular position.

### Setting up the scenario

The facilitator introduced the fiction by explaining that the children would be adopting the role of child psychologists. This technique is known as ‘mantle of the expert’ (Klingberg and Broberg 2007) and sees the children taking on a high status, professional role to solve a problem. Adopting the position of expert affords the children authority over the topic and thus a degree of protection from self-disclosure of their own anxieties. Having reorganised the children’s sitting positions in the class into a more formal setting, the facilitator took the role of a worried social worker and welcomed the pupils to a meeting and explained that their help was needed with a particular child, ‘Katie’, who had developed severe dental anxiety. She explained that Katie was in urgent need of dental treatment, so it was imperative that the psychologists helped resolve her anxiety.

Staying in role as the social worker, the facilitator explained that the ‘experts’ would be able to see the child sitting in a dental waiting room via a two-way mirror. Katie would not be able to see the psychologists, but they should observe her quietly. During this section, an actor-teacher played the role of the child waiting to be called for her appointment. The action was mimed and restricted to a limited set of movements that were repeatable in each school setting. Katie was observed waiting nervously, biting her nails, listening to music on headphones and shrugging off unwanted attention from a parent. Staying in role throughout, the facilitator asked the children what they had observed and asked them to make initial assessments as to the possible causes of the behaviour being displayed.

The fiction focussed entirely on the moments leading up to a visit to the dentist and the anxieties this might provoke. The research team were mindful of not reinforcing negative associations of visiting the dentist but, at the same time, wanted to provide the children with an opportunity to examine the behaviours as observed and what they might signal.

The next section shifted the focus away from working in role to creating the internal world of the character. In small groups, the pupils were asked to create a still image of what they felt Katie was feeling. They were then asked to share these images with the whole class and explain the pictures they had made. Working in a more abstract way allowed the children to operate on a level beyond that of the everyday.

### Probing the problem

The next section of the workshop returned the children to their roles as child psychologists and allowed them the opportunity to decide which other characters associated with this scenario might be able to assist them in their investigation. The children suggested that it would be useful to talk to Katie’s parent, her school friends, teachers and the dentist to see if they could shed light on the situation. The whole research team was involved in playing the various roles suggested by the children and a very rich period of open questioning was enacted by each class. Very few concrete answers or clear reasons for Katie’s anxiety were provided by members of the research team working in role, meaning that the class had to work hard with limited clues. To conclude this section, the social worker extended her thanks to the child psychologists for generously giving up their time and closed the meeting by promising to give them all an update on Katie in due course. Support and advice were provided by the dental team for any child who exhibited any dental anxiety.

### Data analysis

All sessions were audio-recorded and professionally transcribed to be used for data analysis. An inductive thematic analysis at a semantic level was used to explore the data based upon the recommendations of Braun and Clarke (2006). The analysis was undertaken by KG-B (Psychologist and Research Fellow in the design and evaluation of complex intervention). Transcripts were coded using the computer software ‘NVIVO 12’. This requires examining the data to identify common themes or patterns of meaning that reflect the experiences of the participants. Due to the highly interactive nature of the process drama methodology, with both the actors and children actively participating in the generation of ideas through questioning and answering, all the data have been coded and where highly corroborative used to build a meaningful picture of the causes of dental anxiety.

## Results

All children who provided written consent and assent form were included; therefore, in total, 63 out of 89 children aged 7–10 years participated in this study and three sessions of 90-min duration were audiotaped and transcribed.

The data were read and re-read repeatedly to ensure familiarisation with the data and generate ideas for initial codes by KG-B. The entire data set was coded with these codes then being collated to identify significant patterns in the data to form initial themes and sub-themes, which were continually refined to ensure clarity and consistency in meaning.

**Fig. 1:**
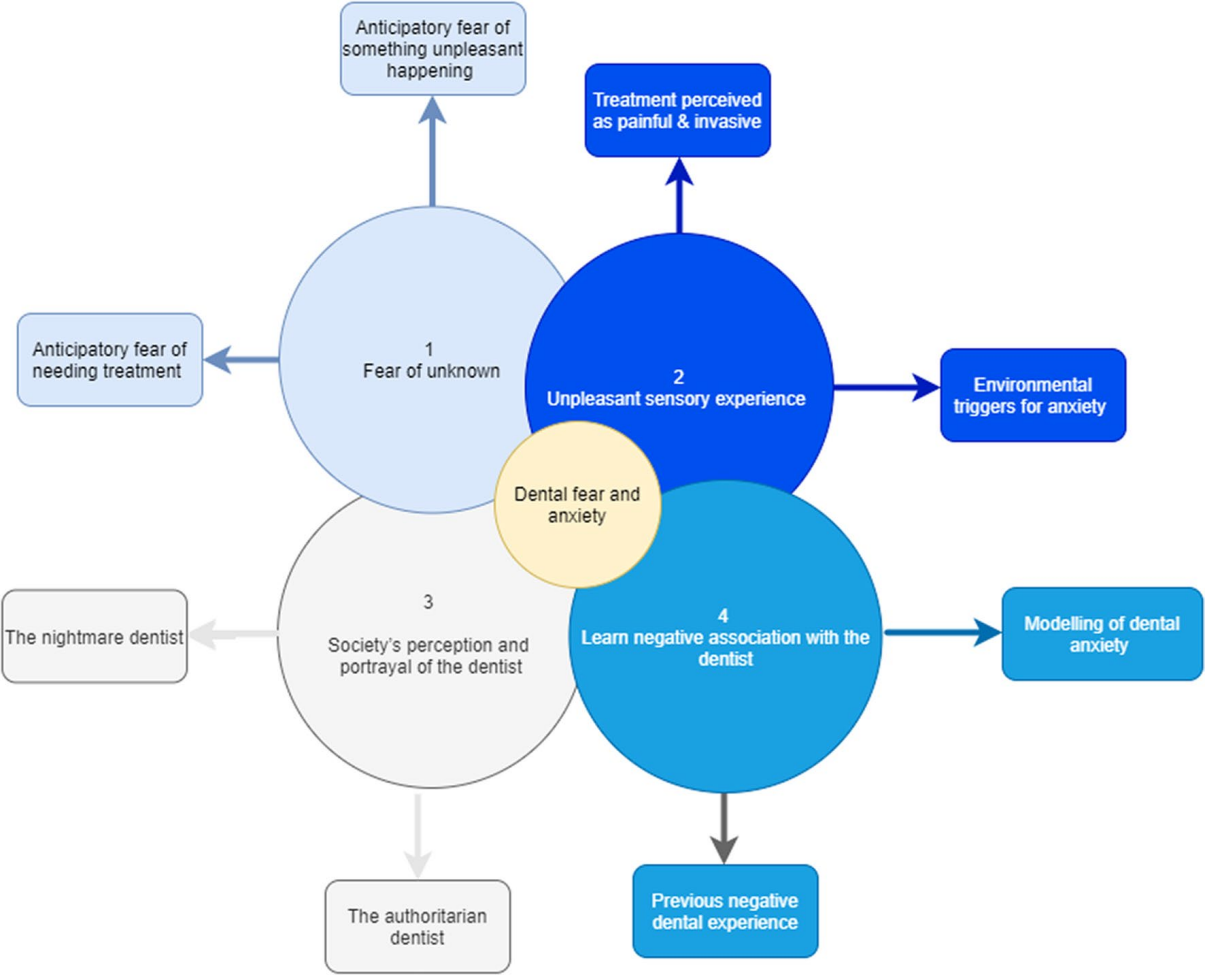
Key themes and sub-themes identified through three sessions of process drama in schools

Four key themes were identified: (1) fear of the unknown; (2) unpleasant sensory experience; (3) society’s perception and portrayal of the dentist; and (4) learnt negative associations with the dentist. Within each theme, two sub-teams were identified, as summarised in Fig. 1. Examples of the stages used to come to final conclusions can be seen in Table 1.

**Table 1:** Example of inductive thematic analysis at a semantic level for the ‘Fear of unknown’ theme

Quotes from transcript	Initial interpretation	Emerging themes	Sub-theme	Key theme
“They’re scared of, well they’re scared of going in here because that they’ve not really… so people aren’t used to it, they don’t really know what’s going on and they’re feeling a bit timid.” “Because sometimes you think that something bad mite [might] happen” “…she’s thinking that he’s going to do something really bad to her.”	Patients possibly fearful and nervous of entering the dental practice and seeing the dentist as they do not know what is going to happen and whatever happens could be potentially unpleasant or distressing	Anticipatory fear and anxiety Loss of control Potential unpleasant experience Fear of the unknown	Anticipatory fear of something unpleasant happening	Fear of the unknown
“I’ve never actually had to have anything done, anything bad done to my teeth so I think it’s being scared of what I might have to have done, or what I might not know what it’s like in case something hurts or is a bit scary.” “I think it’s just the fear of the unknown, not knowing what might happen, if something might hurt, if I might have to have something done to my teeth.”	Patients ‘fear the unknown’ and the possibility of having to receive treatment, particularly dental treatment that could be painful	Anticipatory fear Loss of control Painful dental treatment Fear of the unknown	Anticipatory fear of possibly needing treatment	Fear of the unknown

### Fear of the unknown

1. Anticipatory fear of something unpleasant happening
  Throughout the process drama, both the children and the actors expressed the view that attending a dentist appointment evoked a “fear of the unknown”, relaying that the main character of the drama is scared, because “she doesn’t know what’s happening”. Much of this fear was anticipatory in nature with the waiting room setting acting as a prominent setting for such thoughts and feeling to emerge.
  “I’ve got kind of butterflies in my tummy, when you feel a bit nervous and when you’re kind of going into something that you’re not sure, I don’t know what’s going to happen when I go into the waiting room, when I go into the dentists room, I don’t know how long I’m going to be in the waiting room for, I’m a bit annoyed at my mum because she’s just being annoying, and it’s just kind of lots of things going on in my head it’s quite hard to navigate them all.”
  Although there was a general fear of not knowing what was going to happen, the consensus was that potentially an unpleasant event was going to occur, though what specifically this unpleasant experience was going to be was not always vocalised, and thus appeared to reflect a more generalised loss of control and negative thought process.
  “They’re scared of, well they’re scared of going in here because that they’ve not really… so people aren’t used to it, they don’t really know what’s going on and they’re feeling a bit timid.”
  “Because sometimes you think that something bad might happen”
2. Anticipatory fear of needing treatment
  One possible perceived unpleasant event occurring was the potential need for dental treatment (“Katie is afraid of the dentist because the dentist might take her tooth out”), which may cause pain to the individual.
  “I think it’s just the fear of the unknown, not knowing what might happen, if something might hurt, if I might have to have something done to my teeth.”

### Unpleasant sensory experience

1. Environmental triggers for anxiety
  The dental setting itself was deemed to be an anxiety-provoking environment with the setting possessing a range of negative sensory attributes that acted as triggers for anxiety within the dental environment. These triggers were based around all five senses: touch, sight (“Dentist light”, “seen people scream”), hearing, smell (“Because of the noises and the smell”) and taste. The sound, feel and taste of the dental environment were particularly prominent in creating this unpleasant experience. With regard to the sounds, this related to those made by the operation of dental equipment, most notably the dentist’s drill (“Because Katie can hear all the drills”), as well as the negative reactions of other dental patients vocalised through screaming (“may be that Katie could here [hear] people screaming”). Whether these screams were elicited through dental treatment or fear, and whether the participants had personally experienced hearing patients scream, or this was merely an assumption or perception they had of visiting the dentist was not clear. With regard to taste and touch, this was focused on the actual dental examination of the mouth and teeth, with treatment being perceived as both painful and invasive. Overall, participants expressed a dislike for having the dentist’s fingers and instruments/materials in their mouth, particularly as this can be sometimes an unpleasant taste, with this additionally being seen as an invasion of personal space and privacy.
  “It’s because like when you go to the dentist like sometimes they put something in your mouth and it tastes really weird.”
  “She might not want anyone putting things in her mouth”
  “Actor: So they don’t like them going into their mouths, don’t like other people going into their… it’s quite a private space your mouth isn’t it?
  Participant: Yeah.”
2. Treatment perceived as painful and invasive
  Furthermore, the participants’ expressed a fear of the dental instruments (“…scared of the tools”) and certain dental treatments (“Injections/fillings/teeth pulled out”) with these perceived as potential sources of pain and discomfort.
  “People get anxiety about it because they see them doing it and they don’t like needles because they think it like it could hurt you and stuff.”

### Society’s perception and portrayal of the dentist

1. The authoritarian dentist
  The dentist as an individual was consistently referred to as a key source of dislike (“Maybe it’s not her, like her thoughts about going to the dentist, maybe it’s this specific dentist that she goes to, and she doesn’t like him”) and fear (“So like if she’s like really scared of him she could call the NSPCC”) with a prevailing view that the dentist may be mistreating the patient.
  “Basically I think that she’s sort of, she’s getting like really like upset because probably it’s like that doctor is like doing something like bad behaviour to her, on purpose, and without her mum knowing.”
  “Has your doctor had, did anything to you, like been mean to you or anything like that?”
  In this respect, there were two dominant perceived dentist personas: the strict authoritarian and the nightmare villain. On one level, the dentist was seen as a “rude” and “obnoxious” authoritarian whose role was to chastise and belittle the patients.
  “Katie might be afraid of the dentist because the dentist might make fun of her”
  “…they lecture you”
2. The nightmare dentist
  On another level, the dentist was seen as a nightmarish figure associated with a range of horror-inducing entities and actions. This latter fear-inducing representation of the dentist appeared to be commonly linked with the media’s horror-based portrayal of the dentist in both film (“Evil dentist movie”) and literature (“Scary book”) as a protagonist of evil who relishes inflicting pain on their patients.
  “And also an idea that could, that it didn’t occur to me before but could be reasonable, that Katie’s dental fear, maybe she has quite a really bad nightmare that involved the dentist.”
  “You might have had a nightmare that you were at a haunted Dentic [dentist]”
  “Walked in on a horror film and sor [saw] someone getting toscherd [tortured] and thought that it was dentisted [dentist]”

### Learnt negative associations with dentist

1. Previous negative dental experience
  Similar to the anxiety and fear that can be produced through exposure to our society’s negative media portrayal of the dentist, participants described how negative associations with the dentist could also be learnt through personal experiences and vicariously through significant others.
  “So this one might help a lot, that when she was young did anything like, might have like did she see anything or something like that that might have encouraged her dental anxiety or somehow created it?”
  The participants’ conjectured about how the main character in the process drama may have had a previous “bad experience” when visiting the dentist when she was younger, possibly involving an especially traumatic and/or painful treatment procedure that has left a lasting fear of the dentist that now presents as dental anxiety.
  “When she was younger she might have had multiple teeth taken out”
  “Things have happened in the past”
2. Modelling of dental anxiety
  In contrast, the participants also conjectured that the main characters dental anxiety could have been learnt through knowledge and exposure to her mother’s dental phobia. By observing the negative thoughts, emotions and behaviours experienced by her mother in relation to the dentist and dental treatment both directly and indirectly this could have modelled a pattern of behaviour perceived as an appropriate reaction to the dentist.
  “ Actor: Well she used to come and, come to the dentists with me and watch me having my treatment, so I have had a filling and she watched me having a filling, but I was fine, but maybe that’s, do you think that might be the reason?
  Participant: Possibly.
  Actor: I don’t like going to the dentist myself, so I don’t know, maybe.
  Children: I think it’s because if you’re scared of the dentist then she’s going to be looking at you so she’s going to know that if you’re scared of the dentist then she’s going to be scared of the dentist.
  Actor: I know, I’ve tried not to tell her, and I try not to show it, so I always say it’s fine, you know, let’s go in and I try to be really positive with it, but yeah, I know what you’re saying, I have to try really hard myself.
  Participant: Yeah.”

## Discussion

Dental anxiety is a major issue affecting children’s oral health and clinical management (Wu and Gao 2018). Strategies to overcome dental anxiety are of paramount importance to improve children’s oral health and their experience of dental treatment. Moreover, it may result in a reduction in the number of children requiring dental general anaesthesia due to severe dental anxiety. To overcome dental anxiety a better understanding of the cause is needed. Here we discuss the novel approach to gain understanding of the causes of DFA importantly from the child’s perspective in a non-dental setting. Through application of process drama in a school setting the children are empowered to explore DFA and seek solutions—it has demonstrated that process drama is an effective and fun alternative to questionnaires and other qualitative approaches that have limitations with young children.

School-based drama and theatre-related methods have been shown to have short-term success in health promotion (Joronen et al. 2008). However, there have been a lack of studies with long-term follow-up, which is a common problem in intervention studies (Joronen et al. 2008). Most current drama interventions focus on health knowledge, attitudes and intentions. In addition, most of the reports do not mention the test hypothesis. At present, there appears to be no literature on the use of drama to explore from a child’s perspective the reasons for fear and anxiety related to dentistry.

The results of the current study show that a process drama model presented the drama space as a research laboratory rather than a platform for delivering health messages. In this study, staying in role as the social worker, the facilitator explained that the ‘experts’ would be able to see the child sitting in a dental waiting room via a two-way mirror. Katie would not be able to see the psychologists, but they should observe her quietly. The theatrical convention of placing an imaginary barrier between the observers and the observed allows the facilitator to act as a bridging character, guiding the children’s viewing, eliciting responses and enabling discussion to flow, while the action continues.

Having an adult rather than a child to play the role of Katie ensured that questions and responses from the children could be handled effectively within the drama. The interactive element of process drama requires skilful handling in role to ensure that the enquiry moves forward effectively without breaking the fiction.

The fiction focussed entirely on the moments leading up to a visit to the dentist and the anxieties this might provoke. The dramatic frame provided an opportunity to pay close and deliberate attention to space, movement, gesture, and language that is particularly helpful in determining the relationship between thought, feeling, and action.

Working through the session with “still images” allowed children to work in a more abstract way and to operate on a level beyond that of the everyday. It provided them with the opportunity to access alternative registers of experience and feeling that cannot necessarily be expressed according to logic. Hence, this part of the workshop produced creative, inventive and arguably the most insightful responses to the root cause of Katie’s anxiety, which is described by the present thematic analysis.

The results of the thematic analysis revealed that one of the main reasons for dental anxiety is a fear of the unknown. This was specially highlighted by children as fear of something unpleasant happening or fear of needing treatment. Fear is often considered to be an essential and inevitable emotion, thus providing children with a means of adapting to the stresses of life (Chapman and Kirby-Turner 1999). It is, therefore, normal for children to be afraid of new and potentially unpleasant experiences. It is also reasonable for them to fear a situation, which they may feel harmed them before, such as a painful or traumatic dental experience.

The other key theme identified in the present study was the dental setting being perceived as providing an unpleasant sensory experience. The children mentioned a range of negative sensory triggers associated with dentistry, such as the sound of drill, smell of the dental practice, sight of needles, and taste of dental materials This was in agreement with the study by Nair et al. who reported that dental anxiety typically stems from sensory fear, specifically the sight of, and experience of pain from needles used for injections to deliver local anaesthetic (LA) to numb teeth, and sounds from the dental drill (Nair et al. 2020).

An important part of the child dental experience is the interaction with dental staff, yet this is a relatively under researched topic in terms of dental anxiety. In the current study, one of the main themes that arose was society’s perception and portrayal of the dentist. The children described the dentist as authoritarian or as a nightmarish figure and they linked the dentist’s behavior with media’s horror-based portrayal in films and literature. These findings are similar to the systematic review by Zhou et al. examining the impact of dental care professionals’ clinical behaviours on child DFA. They noted a relationship existed between dental staff behaviour and child DFA, with punishing behaviours associated with high child DFA. Subsequently, to reduce DFA, they suggested that practitioners should adopt an empathic communication style, providing verbal explanation and reassurance (Zhou et al. 2011).

Freeman demonstrated that memories of unpleasant past dental experiences were greater in dentally anxious patients than in non-anxious patients, with dentally anxious patients reporting more experiences of traumatic dental events, thereby indicating that the cause of DFA is more complex than simply a negative past dental experience (Freeman 1991).Their findings are in agreement with the fourth key theme of the present study stating there can be learnt negative associations with the dentist. The results revealed a sub-theme of previous negative dental experience being an important factor in the main character’s dental anxiety. The participant in this study also speculated that the main character’s dental anxiety could have been learnt through observing her mother. In a study by Locker et al. (1999), it was shown that over half of the participants who reported child onset dental anxiety had a parent or sibling who also suffered anxiety about dental treatment. This suggests that, as children, they can indirectly learn their anxious response to dental treatment by observing the behaviour of those around them.

In a qualitative study of DFA in children and adolescents, Gao et al. (2013) analysed videos from YouTube about dentistry. Twenty-seven videos that involved 32 children and adolescents were analysed for the ‘manifestations and impacts of dental fear and anxiety’ and the ‘origins of dental fear and anxiety’. ‘Influence of parents and peers’ emerged as an important point in DFA causation. The present findings add strength to previous research exploring dental anxiety in 5- to 9-year-old children by Tickle et al. who highlighted that dental fear and anxiety can be acquired through adverse conditioning. Both directly through an early negative experience and vicariously through popular culture, family, and friends (Tickle et al. 2009).

Finally, as with all participatory drama activities, ending the session with care provides an important moment of reflection and appreciation of what has been contributed. With a sensitive topic such as dental anxiety, providing clear information, support and advice was an important factor. The participating dental team were available to provide this support and advice at end of each session.

While the study involved only a small number of participating schools and is, therefore, relatively limited in scope, the methodological approach has provided rich data that reveals new insights about the causes of dental anxiety in children. The dramatic framework allowed children to express complex ideas and feelings in ways that other methods such as focus groups, questionnaires and interviews could not achieve. Unlike these other approaches, however, participatory drama processes can never be precisely replicated which produces some variance in results across participating groups. The collaborative nature of process drama means that responses may be shaped by the peer group or other people present. Some children may be unwilling to contribute ideas in such a public setting. However, this study has demonstrated that these limitations can be overcome with skilful facilitation and that some tolerance of variance is necessary when working in this way.

## Conclusion

As dental anxiety is a common problem in children/adolescents worldwide, new strategies to understand the causes of fear and anxiety should be encouraged. Role-play and the use of process drama as a form of enquiry is a novel application to dentistry and can reveal a considerable amount of information on the causes of DFA, importantly captured from the child’s perspective that transcends literacy barriers.
